# Genetic Counselling for Psychiatric Disorders: Accounts of Psychiatric Health Professionals in the United Kingdom

**DOI:** 10.1007/s10897-016-9990-5

**Published:** 2016-07-24

**Authors:** Sian Jenkins, Michael Arribas-Ayllon

**Affiliations:** 1 Oxford Clinical Genetics Service, Churchill Hospital, Headington, Oxford, OX3 7LJ UK; 2 Cardiff School of Social Sciences, Cardiff University, Cardiff, UK

**Keywords:** Genetic counselling, Psychiatry, Schizophrenia, Bipolar disorder, Clinical genetics, Service development

## Abstract

Genetic counselling is not routinely offered for psychiatric disorders in the United Kingdom through NHS regional clinical genetics departments. However, recent genomic advances, confirming a genetic contribution to mental illness, are anticipated to increase demand for psychiatric genetic counselling. This is the first study of its kind to employ qualitative methods of research to explore accounts of psychiatric health professionals regarding the prospects for genetic counselling services within clinical psychiatry in the UK. Data were collected from 32 questionnaire participants, and 9 subsequent interviewees. Data analysis revealed that although participants had not encountered patients explicitly demanding psychiatric genetic counselling, psychiatric health professionals believe that such a service would be useful and desirable. Genomic advances may have significant implications for genetic counselling in clinical psychiatry even if these discoveries do not lead to genetic testing. Psychiatric health professionals describe clinical genetics as a skilled profession capable of combining complex risk communication with much needed psychosocial support. However, participants noted barriers to the implementation of psychiatric genetic counselling services including, but not limited to, the complexities of uncertainty in psychiatric diagnoses, patient engagement and ethical concerns regarding limited capacity.

## Introduction

Genetic counselling is currently available in the United Kingdom (UK) for a variety of disorders and diseases with a substantial genetic component. Concerned with the cause, course, diagnosis and treatment of genetic disorders, genetic counselling is a medical specialty offering information-giving and psychosocial support to affected individuals and their families. Mirroring the broader purpose of genetic counselling (Resta et al. 2006), psychiatric genetic counselling helps individuals and their families with adaption to mental illness, by providing psychosocial support and aetiological information in the context of their own personal and family history. Rather than extending the ‘technological paradigm’ of current psychiatry (Bracken et al. 2012), psychiatric genetic counselling has the potential to create a therapeutic context of empowerment and positive self-identity.

Since the 1930s, estimates from family, twin and adoption studies have shown evidence of a substantial genetic component in psychiatric genetics (Gottesman and Shields 1976). Further studies identifying estimated heritability of schizophrenia, bipolar disorder and depression have strengthened this evidence (Cardno et al. 1999). Burmeister et al. (2008) report that the heritability of Schizophrenia may range between 70 and 85 %, with Bipolar disorder likely to be of a similar heritability at 60–85 %. Other psychiatric conditions reported to be highly heritable include: autism (90 %), attention-deficit hyperactivity disorder (60–90 %) and major depressive disorder (40 %) (Burmeister et al. 2008).

Building on the evidence of heritability, recent advances in genetic technology have developed large-scale approaches of sequencing the human genome searching for common variation between populations. Genome-Wide Association Studies (GWAS) have identified an increasing number of ‘susceptibility’ loci – risk alleles that confer small non-additive genetic effects for a whole range of psychiatric disorders. For instance, the Schizophrenia Working Group for the Psychiatric Genomics Consortium (2014) identified an increasing number of plausible candidate genes implicated in psychiatric disease, with over 100 conservatively defined loci meeting genome-wide significance. These findings provide not only new insights into the aetiology of psychiatric genetics but hold promise for new therapeutic targets.

Today, psychiatric disorders are considered heritable conditions that affect individuals and their families’ worldwide (Gershon and Cloninger 1994; Owen et al. 2000). In the last two decades, genetic research has established the heritability and pathogenesis of psychiatric disorders as multifactorial and polygenic (Laegsgaard and Mors 2008). Genetic risk is thought to be the result of gene-gene and gene-environment interactions (Gottesman, 1991), which is likely to complicate the clinical interpretation of genetic causality (Finn and Smoller 2006).

Despite these aetiological uncertainties, the greatest recognized risk factor for developing schizophrenia and major depression is the presence of a positive family history (Laursen et al. 2005; Austin and Peay 2006). The multifactorial nature of these disorders means a predictive genetic test is never likely to be available for clinical services. However, the absence of genetic testing does not limit the relevance of genetic counselling. Genetic counselling is a skilled practice that focuses on helping families to understand and adapt to the psychological and familial implications of genetic risk (Resta et al. 2006). The focus on ‘client psychological well-being’ is one school of thought that is not reliant on genetic testing to define the scope or efficacy of genetic counselling (Biesecker 2001).

Given the increased familial risks for psychiatric disorders, the potential for genetic counselling has been discussed since the early 1970s (Fraser 1974). The demand for genetic counselling has been established by many studies with up to 70 % of individuals with a family history of schizophrenia expressing an interest in genetic counselling (DeLisi and Bertisch 2006). One study found that unaffected first degree relatives of individuals with a diagnosed psychiatric disorder in Canada perceive genetic counselling to be useful for improving understanding of the disorder and reducing concerns about relative risks (Austin and Honer 2008). However, genetic counselling for psychiatric disorders is not offered in the UK and there is currently no published literature citing demand for such a service. Genetic information or advice is usually provided by the psychiatric team. Although studies have found that psychiatrists consider provision of genetic information as part of their role, less than a quarter considered themselves competent to do so (Finn et al. 2005). This may impact on the efficacy and support provided for these individuals and their families.

Studies have highlighted the benefits of providing education about aetiology (DeLisi and Bertisch 2006; Austin and Honer 2008) for affected individuals and their families by improving knowledge, alleviating anxiety and reducing uncertainty of risk for those with a heightened fear of developing a mental illness (Phillips et al. 2002; Austin and Honer 2007; Hippman et al. 2013). For instance, Rusch et al. (2010) emphasize the importance of discussing causes holistically, where understanding genetic *and* environmental aspects of psychiatric illness is likely to reduce guilt and self-blame. Indeed, models of intervention based on psychotherapeutic interaction are reported to facilitate feelings of empowerment and self-efficacy among clients in the absence of provision of genetic testing (Inglis et al. 2015).

Recent advances in genomics have led to more complex understandings of the genetic contribution to mental illness. These developments are anticipated to increase demand for psychiatric genetic counselling (Austin and Honer 2007) and are expected to drive significant changes in the management and treatment of these disorders (Kaufmann et al. 1996). Despite several studies reporting a demand for genetic testing from both affected individuals and psychiatric clinicians (DeLisi and Bertisch 2006; Hoop et al. 2008a; Hoop et al. 2008b; Laegsgaard and Mors 2008), much caution has been expressed from scientists regarding the clinical validity and utility of new genetic discoveries (Braff and Freedman 2008; Burmeister et al. 2008). Whilst support for genetic testing has been expressed by symptomatic patients (Turney and Turner 2000), there is less agreement on the benefits of presymptomatic testing (Lawrence and Appelbaum 2011).

As large-scale genomics continue to unveil the ‘genetic architecture’ of psychiatric disorders (Gratten et al. 2014), it may become necessary to incorporate forms of psychotherapeutic intervention to accommodate genetic and non-genetic understandings of mental illness. In general, the literature expresses the need for more integration between developments in genetic research and clinical practice.

### Present Study

The present study employs a qualitative approach to explore the potential value and uptake of psychiatric genetic counselling services in the UK. Using a small cohort of psychiatric health professionals, the aim was to uncover in-depth accounts regarding the relevance of genetic counselling to psychiatry.

In light of current advances in genomics, the study was designed to investigate the demand for psychiatric genetic counselling in the UK and the possible benefits and barriers of implementing services in clinical practice. The present study adopts an inductive framework of inquiry to explore the extent to which integration between genetic counselling and clinical psychiatry is desirable or feasible.

## Methods

### Sample and Recruitment

Health professionals working in psychiatry, both from a medical and nursing background, were recruited via invitation emails at Cardiff University and Cardiff & Vale University Trust. Participants completed a short online survey, designed to collect demographic information and assess participants’ prior knowledge of genetics and the genetic counselling service. Participants selected whether they would like to be involved in semi-structured interviews. All participants were required to be over 18 years old, fluent in English, have specific psychiatry training or education, and experience of working with patients affected by psychiatric disorders in a clinical setting. The study was approved by Cardiff & Vale Innovation and Improvement department.

### Data Collection

Individuals who met the inclusion criteria were invited to participate in the study. A link to the online questionnaire was sent to the management structures in the Trust for dissemination by the study gatekeeper.

Thirty two questionnaires were returned. The study gatekeeper estimated that email links were sent to over 100 eligible members of staff, however, due to the method of dissemination this figure cannot be confirmed. The sample included psychiatric nurses (*n* = 16), community psychiatric nurses (*n* = 7) and consultant psychiatrists (*n* = 9). Of the participants who returned the initial recruitment questionnaire, 6 were male and 26 were female. All participants were mental health professionals working for Cardiff & Vale University Health Board Trust aged between 18 and 54.

Of the 12 participants who expressed interest in taking part in the qualitative interviews, two did not respond to further correspondence and one was unavailable for interview until after the study deadline. Nine semi-structured interviews were conducted with psychiatric staff nurses (*n* = 2), a community psychiatric nurse (*n* = 1), consultants psychiatrists (*n* = 2) and consultants psychiatrists with an academic background in psychiatric genetics (*n* = 4). Eight interviews were conducted face-to-face and one by telephone. Informed consent was obtained prior to each interview. All interviews were audio-recorded and transcribed verbatim.

### Data Analysis

Interview transcripts were read and manually coded by the first author and were jointly analyzed with the second author for themes relating to the project’s research questions. The method of ‘thematic analysis’ (Braun and Clarke 2006) used in this study, adopts an inductive approach to qualitative inquiry through an iterative process of reading, coding and identifying recurring patterns and differences within the interview data. The criteria for generating a ‘theme’ is determined by the regularity and intrinsic validity of participants’ responses in terms of offering insights of clinical practice that satisfy the research questions. Themes are treated as *representations* of participants’ current practice and past experience, and thus cannot be regarded as objectively factual. Relevant extracts were selected on the basis of their clear and valid illustrations of themes. Extracts were interpreted by highlighting both ‘surface’ (stated) and ‘latent’ (implied) meanings of participants’ knowledge and experience. In this sense, analysis is concerned with how participants *account* for their knowledge and experience as a discursive practice (Potter and Wetherell 1987).

## Results

### Questionnaire Data

The initial questionnaire provided quantitative data about the demographics and prior background knowledge of the participants regarding psychiatric genetics and genetic counselling (see Table 1 and Table 2).

**Table 1:** Characteristics of questionnaire participants

Variable	*n*	%
Sex		
Male	6	18.75
Female	26	81.25
Age		
18-24	4	22.5
25-34	12	37.5
35-44	6	18.75
45-54	10	31.25
55+	0	0
Knowledge about genetic basis of psychiatric disorders		
No knowledge	0	0
Little knowledge	14	43.75
Some knowledge	14	43.75
Good knowledge	4	12.5
Information about genetics provided during psychiatric training/education		
No information	2	6.25
Little information	14	43.75
Some information	12	37.5
Detailed information	4	12.5
Awareness of the Regional Genetic Counselling Service provided for non-psychiatric disorders		
Yes	8	25%
No	24	75%
Usefulness of a Genetic Counselling service for psychiatric patients		
Yes	18	56.25
No	0	0
Possibly	14	43.75
I don’t know	0	0
Relevance of Genetic Counselling for individuals, and their families, affected by psychiatric disorders		
No relevance	0	0
Little relevance	2	6.25
Some relevance	18	56.25
Very relevant	12	37.5
Likelihood of referring to Genetic Counselling Services		
Yes	16	50
No	2	6.25
Possibly	14	43.75
I don’t know	0	0

**Table 2:** Characteristics of interview participants

Variable	*n*
Sex	
Male	5
Female	4
Age	
18–24	1
25–34	2
35–44	2
45–54	4
Job Title	
Psychiatric Staff Nurse	2
Psychiatric Community Nurse	1
Consultant Psychiatrist (Clinical)	2
Consultant Psychiatrist (Academic)	4

### Interview Data

Participants produced a broad range of accounts on the topic of psychiatric genetic counselling. The themes elaborated below represent the perceived limits of provision and highlight many of the processes and institutional constraints of service-delivery. Each theme is organized into several sub-themes to capture the diversity of participants’ accounts (see Table 3). Analysis revealed the following relevant themes:

1. Demand for psychiatric genetic counselling;
2. Responsibility for genetic counselling provision;
3. Barriers for the service.

**Table 3:** Themes and subthemes with exemplar accounts from the data

Theme/subtheme	Exemplar Account	Professional role
Demand for genetic counselling	“Genetic counselling addresses an area of our patients care that probably is not being addressed at present.”	Psychiatric Nurse
Outcomes of genetic counselling	“It would be useful for patients and their families who are anxious to learn about the disorder and its implications on them as the wider family.”	Clinical Psychiatrist
Genetic counselling as family therapy	“For families as well, I think parents blaming themselves, there is quite a drive at the moment for family therapy within the more acute services.”	Psychiatric Nurse
Effects of genomic advances	“As the sort of genomic explosion has happened all of medicine is genetic.”	Academic Psychiatrist
Responsibility for genetic counselling provision	“It might actually reduce the stigma if it’s done by geneticists rather than psychiatrists. This counselling, it will kind of put the psychiatric disorders under the umbrella of general medical problems.”	Academic Psychiatrist
Genetic counselling as a skilled profession	“A genetic counsellor would be more equipped to deal with risk communication than us, how to say it, what to say.”	Clinical Psychiatrist
Genetic counselling as a specialised service	“To do it properly, it’s very difficult to do without I think a nurse who goes and, takes the time to spend to do the family history and gets records.”	Academic Psychiatrist
Barriers for the service	“They might feel disappointed if we cannot give them any clear information which will probably be in the majority of cases.”	Clinical Psychiatrist
Uncertainty	“Uncertainty is really the bread and butter of the service because when people present, they present quite nebulous and uncertain and so you have got to be able to embrace diagnostic uncertainty.”	Academic Psychiatrist
Limited capacity	“People are cognitively impaired and that’s not to say that they will not understand anything, but it’s to say that the genetic counselling, if they are to receive it, would have to be correctly positioned.”	Academic Psychiatrist
Risk of knowing	“Now saying this could be down to the genes, it’s not just a problem for the patient, you could start worrying everybody else in the family too. Put everyone on high alert.”	Psychiatric Nurse
Patient engagement	“The biggest barrier would probably come from the patients themselves not wanting to engage with the service.”	Psychiatric Nurse

The extracts presented below are labelled to specify the respective role of the participant within psychiatry.

## Demand for Genetic Counselling

In terms of provision in psychiatry, the theme ‘demand for genetic counselling’ produced the most varied accounts. All participants were in agreement that a “specialized service” would be needed in future, but weak patient demand and poor predictability were factors mitigating such a service.

Whilst many participants indicated that psychiatry required more training in genetics, the demand for genetic counselling had not yet come from the patients. In the following extract, a senior academic psychiatrist gives a historical account of previous clinical referrals for psychiatric disorders in the 1990s.

…and the concern I think at the time was that they were getting bombarded with people with cancer who were coming to genetics clinics, adults with common forms of cancer, and I think there was a feeling that there would be a kind of, tidal wave of psychiatric genetics developed, a tidal wave of people coming, and he thought we should be preparing for this. So we set up a clinic in the department of genetics, it was a joint clinic, and we did it for a number of years until he retired and … really since he retired the interest has not been there from the department of genetics much. I should say that we weren’t overwhelmed with people (Male Academic Psychiatrist1)*.*

The high demand for cancer genetics had shaped expectations that similar demand for psychiatric genetics would follow. This prompted an informal collaboration between medical and psychiatric genetics. The respondent alludes to the fragility of this arrangement, implying that interest from “genetics” resided mainly with the collaborator rather than the “department”. However, the unmet expectations of a “tidal wave of psychiatric genetics” implies a stronger reason for why the clinic had few referrals.

Many participants speculated that this low demand related to a priority of environmental rather than genetic factors of psychiatric disorders: “It’s rare for people to think about genetics, issues of genetic risk aren’t top of the list usually” (Male Academic Psychiatrist2). Many affected individuals and their families seek advice and reassurance about more pressing concerns relating to medication and symptoms rather than genetic risk. Even in the context of prenatal counselling for women, who have a higher risk of postpartum psychosis, genetics was a distal concern:

Actually further down the list are other issues that women want to find out and actually quite low down the list is what’s the risk of me passing this illness on to my kid […] because in some respects they are kind of much more proximal problems, aren’t they, like you know, what do I do about my medication now, you know, is this going to make me really ill, these are much more immediate problems (Male Academic Psychiatrist2)*.*

The low demand for genetic counselling is attributed to other priorities, such as giving advice and reassurance about the management of medication and symptoms. In the extract above, the participant is suggesting that issues ‘proximal’ to illness tend to dominate the consultation, which implies that clinical practice is more client-led rather than information-driven.

### Outcomes of Genetic Counselling

Future demand for genetic counselling was often discussed in the context of what genetic counselling can provide for psychiatric patients and their families. Participants frequently cited methods of managing uncertainty and reducing guilt and stigma as key benefits:

Some of them come to me, parents usually, blaming themselves, worrying about the siblings, the brothers, the sisters. I think that’s a key point for your services to our patients (Female Clinical Psychiatrist1)*.*

Here, genetic counselling provides psychological adaptation and reassurance to unaffected family members. The participant implies that psychosocial support mitigates self-blame by presumably foregrounding the complex aetiology of psychiatric conditions. One participant suggested that genetic counselling provides “time” to explain the disorder and reduce anxiety for families:

…the relatives of our patients often have a number of anxieties which stem from lack of understanding, being scared of the unknown you know. I think genetic counselling may be just what they need, somebody to talk them through the ins and out of the disorder, we don’t tend to spend enough time covering it (Female Psychiatric Nurse2).

There is a sense in which genetic counselling offers aetiological information to relieve family anxiety, which positions the service in the role of providing additional family support. The statement “we don’t tend to spend enough time covering it” implies that psychiatric nurses *could* perform this role but lack the resources to do it properly.

### Genetic Counselling as Family Therapy

Similarly, there was a notion that genetic counselling could be utilized as an additional “therapeutic tool” for both patients and their families. The availability of a service which deals with at risk relatives as well as affected individuals was identified by participants as a key part of the service:

If you’ve got a spouse of somebody that’s had a really severe psychosis, I’d imagine they would want that information. You can imagine that genetic counselling would be really useful in that, which is something that isn’t offered widely but there probably would be uptake for (Male Psychiatric Nurse2)*.*

The participant offers a hypothetical account of the benefits of genetic counselling for unaffected families, though it is unclear what kind of “information” this entails. Markers such as “would imagine”, “can imagine” and “probably would” suggest a vague understanding of genetic counselling, though, in a positive sense, it provides an alternative space for family support: “somewhere else to discuss and express their concerns”. This was supported by another participant who expressed that families often do not like to raise concerns about their own well-being in the affected individuals’ consultations with the psychiatric team.

That a relative can go to see a counsellor independently, not as part of their relatives care, sometimes they think they can ask about themselves and the impact on them, we don’t get around to it, they like to concentrate on the treatment of their kid or parent or whoever it may be. They don’t want to eat into their time (Female Clinical Psychiatrist2)*.*

Genetic counselling is value-adding in terms of providing families an additional space of support. Participants recognized the unique aspect of clinical genetics in its care of families rather than individuals; it provides a private and supportive space to minimize the psychological “impact” of mental illness. Again, the reference to “time” implies that genetic counselling is perceived as an extension of therapeutic support in the context of limited professional resources.

### Effect of Genomic Advances

Most participants demonstrated an awareness of genomic advances within the field of psychiatry, though some discordance was observed regarding their implications for future clinical practice. Those involved in psychiatric genetics expressed willingness that genetic research should: “inform clinical psychiatry, part of that would be genetic counselling”:

We have got to the stage now where we have much more knowledge, and we should start to think about it in a way, setting up a clinic, a clinical application for it (Male Academic Psychiatrist1).

Though transferring genetic research to the clinical setting is desirable, there is also a sense in which “the next stage” of clinical translation is unclear. Ethical issues regarding early diagnosis and prevention or unnecessary medicalization warrant caution about translating genetic discoveries from the laboratory to the clinic:

I don’t think people have really got their heads around the predictive abilities of the genetic knowledge that we have yet, I think it’s still too, too early to say and… I think the position is still… there’s a long way to go before we can use genetics to predict risk in the population and everybody’s really cautious about doing it (Male Academic Psychiatrist1)*.*

Although they had not experienced explicit requests for genetic counselling, participants reported an *expectation* that affected individuals and their families would benefit from such a service. While all the participants attested to the benefits of a patient-centred approach of genetic counselling, non-genetics professionals conveyed a sometimes vague understanding of its specialization, implying that it provided “additional” care and support for families of affected individuals. Lastly, a sense of caution was raised in relation to explicitly linking genetic services to testing.

## Responsibility for Genetic Counselling Provision

Many participants deliberated over professional responsibility for psychiatric genetic counselling provision. Some concluded that the best arrangement would be a joint collaboration of expertise rather than allocating sole responsibility to either clinical genetics or psychiatry. Several issues were raised regarding the suitability of both services to provide genetic counselling for psychiatric disorders which form the basis of further subthemes.

### Genetic Counselling as a Specialized Service

For those working in psychiatric genetics, genetic counselling was perceived as a service specific to medical genetics, and thus separate from academic psychiatry. In the extract below, the senior academic psychiatrist is contrasting genetic counselling with their own genetics clinic:

We run our own clinic, tertiary referral clinic, we get some patients who wish to have genetic information, we don’t call it genetic counselling, we don’t believe we’re doing it formally in that sense, but we offer genetics advice. So we do occasionally have people coming [with a] family history of dementia, family history of psychotic disorders and sometimes families with high densities of a number of different things. So, you know, it is very much a question of seeing the person and trying to get the records, but without… to do it properly, it’s very difficult without I think a nurse who goes and takes the time to do the family history (Male Academic Psychiatrist1)*.*

The participant makes a distinction between genetic counselling as a “formal” service and the kind of clinic they offer, which centres on “genetic advice”. Genetic advice-giving is oriented to the collection of information about family history, the gathering of records that may assist in making risk assessments. However, gathering information from families is a specialized skill requiring both “time” and interactional expertise “to do it properly”. From an academic perspective, genetic counselling is perceived as a formal specialism that needs to be developed within psychiatric genetics:

I guess it’s a professional specialism, you know… particularly, medical genetics is, but also the nurses are trained in ways that we’re not. I guess we don’t have the precision… most of the cases we see are much more murky. I think, there is a need to have a more formal set up. And we have thought about that […] So we may well try and develop a formal clinic with a trained nurse, because… I think we are identifying now these cases and families where they have quite highly penetrant mutations, which confusingly predispose to a range of different psychiatric disorders... but mainly neurodevelopmental so, you know, these are quite complicated families to advise and I think there is going to be a need to develop the expertise to deal with that (Male Academic Psychiatrist1)*.*

The perception of genetic counselling as a “professional specialism” highlights a unique form of training not offered in the psychiatric genetics clinic. The participant attributes this to psychiatric disorders lacking the “precision” of phenotypes seen in medical genetics. The formality of genetic counselling is seen as offering a more rigorous approach to collecting complex case histories and advising families in ways that may support the mutual interests of academic psychiatry and genetic counselling. Interestingly, this expertise is not imagined as a single professional role, but a collaborative relationship between psychiatrists and “trained nurses”.

### Genetic Counselling as a Skilled Profession

Many participants recognized that genetic counselling embodied a unique set of skills suited to dealing with the genetics of psychiatric disorders. Only one participant expressed concern that genetic counsellors do not feel equipped to deal with psychiatric disorders in their current practice, however, all participants were in agreement that genetic counselling is a “skilled profession”, and better equipped to communicating genetic risk than psychiatric services.

A common observation was that psychiatry did not receive training in the provision of genetic risk information; many saw this as a skilled practiced belonging to “clinical genetics”:

I don’t think psychiatrists are trained in that necessarily. No less than any other doctors I’m sure… so you’d imagine that clinical geneticists and people who regularly do genetic counselling are far more skilled in that sense, obviously trained for it and certainly that’s not the case for… people who have gone through sort of generic medical training or even speciality training (Male Academic Psychiatrist3).

Genetic counselling involves a “far more” specific set of skills than what is available in the “generic” training of psychiatrists. In this sense, there is very little difference between the training of doctors and psychiatrists. These contrasts serve to distinguish genetic counselling as an established sub-discipline from which psychiatry can benefit.

In a similar vein, the senior academic psychiatrist also highlighted the generic nature of communicating recurrence risk to families, which implies that genetic counselling is more than merely information-giving:

I think the giving of information is a fairly generic skill that any doctor or nurse should be able to master… it’s trying to formulate what information to give, I think that may require more training than the average consultant psychiatrist can do… So for example, the psychiatrist should know the recurrence risks in different classes of relative but then you start to say, well actually I’ve got two, my uncle Fred had bipolar disorder, what does that mean, are my risks different and most people will be thinking, this is beyond my level of expertise, and actually they are very difficult questions to answer. So that’s where I think you might need a more specialised service (Male Academic Psychiatrist1).

Where communicating recurrence risk is a transferrable skill that “any doctor or nurse should be able to master”, genetic counselling is a “specialised service” more suited to dealing with complex information-giving, especially when multiple family members are affected. However, it is interesting to note the absence of emotion in this description as an important component of risk communication.

Whilst many participants acknowledged that genetic counselling was a skilled practice of communication, only one participant emphasized the distinct benefits this may have for patients:

Combining the expertise and knowledge of psychiatry and genetic counselling will provide our patients with genetic risk information that will allow personal empowerment, that’s our goal, to empower our patients to make informed decisions (Female Psychiatric Nurse1).

This emphasis on patient empowerment was not articulated by other participants, though it was implied that communicating risk and uncertainty had benefits for individuals and their families. Here, the psychiatric nurse suggests that combining the expertise of psychiatry and genetic counselling is value-adding: that communicating complex risk information is not merely edifying or reassuring, but it enables patients to make difficult decisions.

From the perspective of psychiatry, then, we see a glimpse of how psychiatric genetic counselling may be organized in the future. Genetic counselling implies a collaborative arrangement in which emotional work and family history-taking is combined with the diagnostic and medical expertise of psychiatry. This suggests that the provision of psychiatric genetic counselling would be more suited to a separate sub-discipline, with trained genetic counsellors developing expertise in psychiatry.

## Barriers for the Services

As a result of the service not being currently available, barriers for referral of genetic counselling services were discussed by participants. Four main barriers were highlighted.

### Uncertainty

Uncertainty was discussed in relation to two contexts of service provision. Firstly, the difficulties in providing a firm diagnosis for those affected by psychiatric disorders would be a barrier for referrals to the service. Without a definite diagnosis, information and risk communication lacks precision.

It’s often quite difficult to make a precise diagnosis in the relative which then alters the precision of what you can do (Male Academic Psychiatrist4)*.*

Arguably, precision of diagnosis is necessary for information to be useful to families, though the literature does not necessarily support this assumption (Inglis et al. 2015). Nevertheless, the participant suggests that genetic counselling would be of limited use in the absence of diagnostic precision.

Secondly, factors predisposing to psychiatric disorders are essentially probabilistic rather than deterministic. Uncertainty surrounding the risks of susceptibility loci and pleiotropy of genes (where one gene can influence multiple phenotypic traits) reduces the clinical utility of information provided in a genetic counselling consultation.

Genetics in this sense isn’t determinate, just because he’s got that genetic risk doesn’t mean that he’s going to get the condition (Male Academic Psychiatrist2)*.*

The complex aetiology of psychiatric disorders encompasses both non-genetic factors as well as genetic factors associated with several disorders. However, the uncertainty arising from probabilistic risk can have positive consequences of avoiding a fatalistic impression of disease. Thus, while the inherent uncertainty of genetic risk is a barrier for the service, it also presents important opportunities to reassure patients.

### Limited Capacity

Many participants cited the ethical concern of limited capacity or autonomy of clients affected by a psychiatric condition. Acting upon genetic information relies on the ability of patients to give informed consent or engage in shared decision-making:

I think I agree one needs to be an adult to make those decisions and there’s the question of capacity, people who are already ill, do they understand what they are asking for and what it would mean (Male Academic Psychiatrist1).

Adapting information to patients with cognitive impairment is both a challenge and a skill which seems to be a more prevalent concern for clinical psychiatry.

They come to us on average a standard deviation, at least sort of 15 IQ points below the healthy population, so straight away you’ve got to gear your consultation and communication to somebody in that sense (Male Academic Psychiatrist2)*.*

From this description of patient population, capacity to provide consent is evidently variable. In such cases of reduced autonomy, communicating complex risk information may be neither desirable nor achievable in the consultation, in which case a genetic counselling service would need to balance the “best interests” of clients with issues of capacity.

### Risk of Knowing

Among the non-genetic professionals, there were concerns that knowing one’s genetic risk may intensify stigma and blame, which is contrary to the view that genetic explanations may actually reduce stigma and blame (Austin and Honer 2005). One participant described the difficulty of discussing genetic risk associated with psychiatric disorders as “walking a tight rope”:

Most of my patients aren’t aware of the genetic implications of their disorder. They don’t think their families are at risk. It could open up a can of worms (Female Psychiatric Nurse3)*.*

The expression “can of worms” implies that the personal and familial consequences of knowing one’s risk may generate more harm than good. Again, balancing the patient’s autonomy and their best interests would seem to be particularly relevant to clinical psychiatry. The psychiatric nurse alludes to the scenario that attempting to solve one problem might inadvertently create more problems.

The risk of knowing is a danger, but if it’s done in a way that protects the individual in a supportive way so they know the help is out there. It’s probably harder than a lot of other conditions because there is no sort of X Y Z, this is how it’s all going to pan out because everybody is different (Female Psychiatric Nurse1).

The challenge posed by this health professional is balancing a paternalistic approach of withholding information with psychosocial support. Genetic information has significant implications for the individual and their family, however, it should not hinder the individual’s right to be aware of his/her genetic risk.

### Patient Engagement

Patients’ unwillingness to cooperate in genetic counselling services is also indicative of potential barriers for provision. For instance, the stigma related to psychiatric disorders may reduce uptake for psychiatric genetic counselling:

People often tend to feel more ashamed in psychiatry for carrying something that causes [a] psychiatric disorder, so a stigma. They might not want to come because they might be told “you’ve got something” (Female Psychiatric Nurse2).

And one psychiatrist stated that patients do not always attend appointments because the service itself is stigmatized:

Psychiatric patients often have a vendetta against our health service, they’ve been let down by it many times in the past. They don’t engage. We have trouble getting patients to attend appointments here, it’s not the same as having a physical complaint, they’re used to it, and they think there’s nothing we can do to help, so why bother (Female Clinical Psychiatrist1).

In this candid account, the participant highlights several barriers owing to the poor relationship between patients and mental health services. Patients with chronic histories of illness may not see the benefit of genetic counselling by virtue of its association to existing services.

Patient engagement is clearly a factor that may affect the success of provision. As this study has highlighted, respondents perceived that issues of genetic risk are not a major priority for many affected individuals; this, in addition to the barriers identified here, may continue to hinder the implementation of a genetic counselling service.

## Discussion

This is the first study to explore accounts of mental health professionals with respect to future service provision of psychiatric genetic counselling in the UK. Participants noted that there was no current demand for psychiatric genetic counselling from the patient group and their families as the aetiology of these disorders are not well understood. From the initial recruitment survey, only 25 % of participants were aware that a genetic counselling service was available for non-psychiatric disorders, highlighting that awareness of the genetic counselling service is limited among other healthcare providers. However, DeLisi and Bertisch (2006) found that 70 % of individuals with a family history of schizophrenia in a New York study would seek referral to genetic counselling, though few have received genetic services. This may suggest that the awareness and interest for psychiatric genetic counselling as a future service is growing. Further study on the perspectives of affected individuals and their families is needed in the UK to confirm whether there is service demand.

Provision of psychiatric genetic counselling services was advocated by the participants of this study as a unique “specialism”. Communicating genetic risk is a “skilled profession” capable of reducing feelings of anxiety, guilt and stigma. Only one participant identified such a service as “empowering” patients to make informed decisions. These findings are consistent with the reported aims and outcomes of genetic counselling more broadly, and fit the model of empowerment proposed by McAllister et al. (2011).

These outcomes have been reported by previous studies into the provision of genetic counselling for mental health in Canada (Austin and Honer 2007; Austin and Honer 2008: Hippman et al. 2013). Recently, Inglis et al. (2015) utilized the Genetic Counselling Outcome Scale (GCOS) (McAllister et al. 2011) to assess specialist genetic counselling provision, with individuals and their family members reporting a significant increase in feelings of empowerment one month post genetic counselling.

Professionals’ accounts of the future of psychiatric genetic counselling centred on the advances made in recent years in genomic research. Consistent with other studies, participants formulated *expectations* these such advances will have an impact on the demand for genetic counselling in the future (Turney and Turner 2000; Austin and Honer 2007). However, academic psychiatrists were particularly cautious about the clinical application of genetic research in light of eugenic concerns regarding the history of psychiatric genetics (Gottesman and Bertelsen 1996; Schulze et al. 2004). Some emphasized apprehension when discussing genetic counselling in the context of genetic testing. Although previous studies (Laegsgaard and Mors 2008; Lawrence and Appelbaum 2011) found that psychiatric care providers and affected individuals and their families advocated the use of genetic testing, this was not supported in this study. The absence of a genetic test in psychiatry was significant to the relevance of genetic counselling in these disorders. Some participants indicated that genetic counselling went hand-in-hand with genetic testing, that without the availability of a genetic test genetic counselling could only provide uncertain risk information. A study looking at the provision of psychiatric genetic counselling in Canada, however, found that positive outcomes for genetic counselling were not reliant on genetic testing (Inglis et al. 2015). Genetic counselling for psychiatric disorders can provide information on the cause and course of psychiatric disease in addition to discussing medical management and environmental risk factors, and thus, the absence of an informative genetic test need not be a barrier for referral to the service.

No previous studies have explored whether psychiatric genetic counselling would be better positioned within the present structure of psychiatric services or genetic services in the UK. Participants attributed the current shortfall of genetic counselling provision to existing demands of workload as well as limited understanding of psychiatric genetics by those in the profession. This is consistent with the initial recruitment survey data which found that only 12.5 % of participants expressed at least a ‘good knowledge’ of the genetic basis of psychiatric disorders. It is likely that these numbers reflect the academic psychiatrists who took part in this study, highlighting that currently only specialised research centres have expertise in psychiatric genetics. This is in keeping with a previous study by Finn et al. (2005), which found less than 25 % of psychiatrists felt competent to provide genetic information.

From these data, we can see that knowledge of the genetic basis of psychiatric disorders correlates to the role of the health professional and the subsequent training or education they have received. Participants who reported that ‘no information’ was provided in their psychiatric training were both 45–54 years of age and psychiatric nurses, suggesting that psychiatric nurses who received their training prior to the advent of genomic research are less likely to be aware of the significance of genetics for psychiatric disorders. As most genetic discoveries have been made in the last 20 years (Andreasen 2005), the quality of information provided in psychiatric training is likely to have changed substantially. Nevertheless, the limited knowledge of genetics among psychiatric health professionals may itself serve as a barrier to psychiatric genetic counselling. The aetiology of psychiatric disorders was found to be of limited importance in clinical practice, with consultations oriented to issues of diagnosis, medication and prognosis. Subsequently, without a focus on the biological model of psychiatric disorders, professionals may not see the need to refer patients to the service.

Although psychiatric services deem that a specialized service would be more appropriate to take responsibility for psychiatric genetic counselling, concern over the views of genetic counsellors’ towards mental health as a field is not exclusive to this study (Feret et al. 2010). A recent workshop on ‘Psychiatric Genetics for the Genetic Counsellor’ led by Dr. Jehannine Austin and Kevin McGee at Bournemouth University, attracted over 20 UK genetic counsellors, all with a special interest in this sub-discipline. This may suggest a sea change in attitudes of genetic counsellors’ towards psychiatry.

Although UK genetic counselling services do not routinely see psychiatric disorders in clinic, several conditions counselled by clinical genetics confer a risk to psychiatric disorders, for example, 22q11.2 deletion syndrome and Huntington’s Disease (HD). Martin et al. (2012) expressed that genetic counsellors are reluctant to disclose the risk of psychiatric disorders in these conditions, with a lack of understanding of psychiatric disorders and their associated stigma making discussions regarding psychiatric risk challenging. Further studies to explore the perspectives of genetic counsellors are needed to assess the feasibility and suitability of service provision in the UK. Although this concern was raised in the study, all participants were in agreement that a specialised service was valued for the proper delivery of a psychiatric genetic counselling service.

Barriers to referring individuals and their families to the service were described in detail by participants, highlighting areas of concern over future service-delivery. Over 90 % of the initial survey participants felt they would refer to an available genetic counselling service in future, over 90 % felt the service had some relevance and over half of participants reported the service to be useful and informative to their patient group. This suggests that these barriers would not hinder referrals, or the validity of the counselling provided. However, issues with patient engagement were raised that would affect the success of the service despite patient referrals.

The concept of ‘uncertainty’ played two roles in this study. Diagnostic uncertainty was presented as a barrier for referral to the genetic counselling service due to the difficulty in providing accurate psychiatric diagnoses in a field where many conditions have an overlapping phenotype. Concern was expressed over the validity of information genetic counselling could provide in light of this diagnostic uncertainty. However, uncertainty was also described as a tool for reassurance, which may have positive consequences for avoiding a fatalistic impression of psychiatric disorders. Uncertainty is not unique to psychiatric genetics. Genetic counsellors are well-versed in these discussions in the field of cancer genetics and the increasing identification of variants of unknown significance (VUS). The impact of uncertainty in the context of psychiatric diagnoses and psychiatric genetics should be explored further.

Ethical considerations’ regarding the provision of psychiatric genetic counselling is an area that requires further consideration. Participants were especially concerned with the limited or variable capacity of affected individuals and their ability to understand complex information on which subsequent decisions are made. A challenge presented by the participants for the service was the ability to adapt communication to enhance informed consent without increasing psychosocial burden. However, many genetic conditions currently seen in clinical genetics also have a significant effect on cognitive ability, often associated with learning disabilities, and therefore should not present a new challenge to genetic counselling.

### Study Limitations

Four participants in this study have a background in psychiatric genetics and therefore have a greater knowledge of genetics than is typically found among clinical psychiatrists. The range of expertise in the sample reflects the variablity of knowledge observed in this study.

Due to external constraints of completing the research within the framework of a supervised Masters Dissertation, data collection did not achieve thematic saturation, and therefore provides a limited insight into the accounts of psychiatric health professionals. Further exploration of individuals working in clinical psychiatry is vital to reveal a broader range of perspectives.

### Research Recommendations

This study is the first of its kind to explore healthcare providers’ accounts of psychiatric genetic counselling in the UK. Further exploratory research is needed with different and larger cohorts to examine the interface between healthcare professionals and patients in order to provide recommendations for the future of psychiatric genetic counselling. Further research should include exploration of patient perspectives on the value of psychiatric genetic counselling to identify the compatibility of patient and professional perspectives.

## Conclusions

Although demand for psychiatric genetic counselling has not been formally voiced in the UK at present, the accounts of psychiatric health professionals indicate that such a service would be useful and desirable. Advances in the identification of susceptibility loci for psychiatric disorders may have significant implications for genetic counselling in clinical psychiatry, whether or not these discoveries lead to genetic testing.

Psychiatric health professionals describe clinical genetics as a skilled profession capable of combining complex risk communication with much needed psychosocial support. However, they envisage that the possibility of such a service is confronted with a range of barriers and challenges including, but not limited to, the complexities of uncertainty in psychiatric diagnoses, patient engagement and ethical concerns regarding reduced patient autonomy.

## Appendix 1

### Semi-structured Interview Guide

1. Can you tell me about your experience of genetic counselling in your current role?
2. Can you tell me some specific stories about your experience?
3. What kind of families or individuals do you see?
4. Have you ever been directly approached for genetic counselling?
5. Can you take me through the procedure of how you might describe and explain genetic risk to families?
6. Do counselling protocols change significantly depending on the disorder, i.e. does severity condition change the way you explain or perform counselling?
7. How important are environmental factors in discussing risk?
8. What are some of the ethical or practical challenges of (genetic) counselling for psychiatric disorders?
9. Do you think recent scientific advances in genetics and genomics have changed (or need to change) the way genetic counselling services are offered for psychiatric disorders?
10. How do you see genetic counselling of psychiatric disorders developing in the future (in the UK)? Is there demand?
11. How might genetic counselling services be adapted to provide psychiatric genetic counselling?
12. If someone had to write about the ethical issues surrounding genetic counselling on psychiatry what would you identify as the main issues?
